# Draft Genome Sequences of *Vibrio alginolyticus* Strains V1 and V2, Opportunistic Marine Pathogens

**DOI:** 10.1128/genomeA.00729-15

**Published:** 2015-07-02

**Authors:** Daniel Castillo, Paul D’Alvise, Panos G. Kalatzis, Constantina Kokkari, Mathias Middelboe, Lone Gram, Siyang Liu, Pantelis Katharios

**Affiliations:** ^a^ Marine Biological Section, University of Copenhagen, Helsingør, Denmark; ^b^ Department of Systems Biology, Technical University of Denmark, Kongens Lyngby, Denmark; ^c^ Institute of Marine Biology, Biotechnology and Aquaculture, Hellenic Centre for Marine Research, Former American Base of Gournes, Crete, Greece; ^d^ BGI Europe A/S, Copenhagen, Denmark

## Abstract

We announce the draft genome sequences of *Vibrio alginolyticus* strains V1 and V2, isolated from juvenile *Sparus aurata* and *Dentex dentex*, respectively, during outbreaks of vibriosis. The genome sequences are 5,257,950 bp with a G+C content of 44.5% for *V. alginolyticus* V1 and 5,068,299 bp with a G+C content of 44.8% for strain V2. These genomes provide further insights into the putative virulence factors, prophage carriage, and evolution of this opportunistic marine pathogen.

## GENOME ANNOUNCEMENT

*Vibrio alginolyticus* is a Gram-negative bacterium and an important opportunistic pathogen for marine organisms (1). This bacterium is associated with epidemic vibriosis, which results in high mortality for cultured marine animals, including fish (2), shellfish (3) and shrimp (4). In addition, *V. alginolyticus* can be pathogenic in humans and can lead to otitis and wound infections after contact with *V. alginolyticus*-containing seawater (5). We report the draft genome sequences of *V. alginolyticus* strains V1 and V2, which were isolated from farmed juvenile *Sparus aurata* and *Dentex dentex*, respectively, during two separate vibriosis incidences in Crete, Greece.

*V. alginolyticus* strains V1 and V2 were grown in Luria broth (Mo Bio, 12106-05) supplemented with NaCl 1.7% overnight at 22°C with agitation. Genomic DNA was extracted using the QIAamp DNA minikit (Qiagen) according to manufacturer’s protocol. A sequencing library was prepared using an Illumina HiSeq platform (BGI, China) with pair-end read sizes of 100 bp. A total of 9,374,944 paired-end reads for strain V1 and 10,880,660 paired-end reads for strain V2, were used for *de novo* assembly in Geneious version 7.1.7 (http://www.geneious.com). Short and low-coverage contigs were filtered out, resulting in a set of 80 with an average coverage of 129× (*N*_50_, 265 kbp), and 33 contigs with a coverage of 137× (*N*_50_, 340.4 kbp) for *V. alginolyticus* strains V1 and V2, respectively. Annotation was performed using the NCBI Prokaryotic Genome Automatic Annotation Pipeline (PGAAP) (6). Additionally, the genomes were analyzed on the Rapid Annotation using Subsystems Technology (RAST) server (7). Genome comparison was achieved using Mauve version 2.4.0 (8). Acquired antibiotic resistance genes were identified using ResFinder version 2.1 (9), virulence factors by virulencefinder version 1.2 (10), and prophages by PHAST (11).

The final assembly for *V. alginolyticus* strain V1 had a total length of 5,255,839 bp and a G+C content of 44.5%. Genome annotation resulted in 4,614 coding sequences (CDSs), 67 tRNAs, 59 pseudogenes, and 5 rRNAs. *V. alginolyticus* strain V2 had a total length of 5,068,299 bp and a G+C content of 44.8%. Genome annotation resulted in 4,448 CDSs, 77 tRNAs, 42 pseudogenes, and 4 rRNAs. Prophage screening for *V. alginolyticus* strain V1 displayed four phage-related sequences of 9.3, 14, 16.5, and 34.9 kb. Similarly, *V. alginolyticus* strain V2 had four phage-related sequences of 8.6, 11.5, 20.7, and 30 kb. *V. alginolyticus* strain V1 possesses two unique regions associated to drug resistance and transport of iron. For both strains, putative virulence factors were identified with functions such as adhesion and destruction of tissues (collagenases, arylsulfatases, proteases, and hemolysin), ABC-type transport systems (spermidine, putrescine, iron), and toxins (RTX, YafQ), which may facilitate specific pathogenicities of *V. alginolyticus* in different environments. No antibiotics resistant genes were detected.

Thus, these genome sequences can facilitate future comprehensive comparison and phylogenetic analyses aiming toward more efficient control of this opportunistic pathogen.

### Nucleotide sequence accession numbers.

The draft genome sequence of *V. alginolyticus* strain V1 can be accessed under the GenBank accession number LCUM00000000 and *V. alginolyticus* strain V2 under the accession number LCSG00000000. The versions described in this paper are LCUM10000000 and LCSG10000000, respectively.
